# Sounds of COVID-19: exploring realistic performance of audio-based digital testing

**DOI:** 10.1038/s41746-021-00553-x

**Published:** 2022-01-28

**Authors:** Jing Han, Tong Xia, Dimitris Spathis, Erika Bondareva, Chloë Brown, Jagmohan Chauhan, Ting Dang, Andreas Grammenos, Apinan Hasthanasombat, Andres Floto, Pietro Cicuta, Cecilia Mascolo

**Affiliations:** 1 Department of Computer Science and Technology, University of Cambridge, Cambridge, UK; 2 ECS, University of Southampton, Southampton, UK; 3 Department of Medicine, University of Cambridge, Cambridge, UK; 4 Department of Physics, University of Cambridge, Cambridge, UK

**Keywords:** Biomarkers, Epidemiology

## Abstract

To identify Coronavirus disease (COVID-19) cases efficiently, affordably, and at scale, recent work has shown how audio (including cough, breathing and voice) based approaches can be used for testing. However, there is a lack of exploration of how biases and methodological decisions impact these tools’ performance in practice. In this paper, we explore the realistic performance of audio-based digital testing of COVID-19. To investigate this, we collected a large crowdsourced respiratory audio dataset through a mobile app, alongside symptoms and COVID-19 test results. Within the collected dataset, we selected 5240 samples from 2478 English-speaking participants and split them into participant-independent sets for model development and validation. In addition to controlling the language, we also balanced demographics for model training to avoid potential acoustic bias. We used these audio samples to construct an audio-based COVID-19 prediction model. The unbiased model took features extracted from breathing, coughs and voice signals as predictors and yielded an AUC-ROC of 0.71 (95% CI: 0.65–0.77). We further explored several scenarios with different types of unbalanced data distributions to demonstrate how biases and participant splits affect the performance. With these different, but less appropriate, evaluation strategies, the performance could be overestimated, reaching an AUC up to 0.90 (95% CI: 0.85–0.95) in some circumstances. We found that an unrealistic experimental setting can result in misleading, sometimes over-optimistic, performance. Instead, we reported complete and reliable results on crowd-sourced data, which would allow medical professionals and policy makers to accurately assess the value of this technology and facilitate its deployment.

## Introduction

Since its outbreak in early December 2019, over 169 million cases of the novel coronavirus disease have been reported, including 3.5 million deaths. Researchers and scientists have made considerable strides in developing treatments and vaccines for COVID-19, and effective and easily accessible tests have been key to trace infected people quickly. Currently, the most commonly used and first-line diagnostic tool for COVID-19 is the reverse transcription polymerase chain reaction (RT-PCR) assay to detect the presence of viral ribonucleic acid (RNA) from swab samples [1, 2]. RT-PCR tests are highly sensitive in laboratory setting (over 95% diagnostic sensitivity and specificity), however, they have been found to perform differently in commercial kits, with sensitivity ranging from 75 to 100%, and in the worst case reaching as low as 38% [3–5]. Moreover, the sample analysis process is involved, time-consuming, and limited to approved laboratories with highly-trained staff, leading to limited testing capacity and failing to meet the rapid increase in demand. Computer tomography (CT) scans are gaining popularity for COVID-19 diagnostics in some countries, e.g. China [6]. However, this method had not been widely adopted worldwide due to many doctors remaining sceptical about the reported high sensitivity [7]. In addition, CT scanners are specialised and expensive equipment suitable only for medical centres with trained staff for its operation. For inpatients, on top of a high price tag for a single scan, patient transport to and from the scanner requires to break the isolation, which significantly increases the infection transmission risk. It is crucial that the pandemic response overcomes the limitations from RT-PCR and CT to timely test on a massive scale. This requires fast, affordable, sustainable and effective testing methods, which can be repeated over time by individuals to track progression. This would help contain the current spread but also suppress resurgence and minimise health risks.

Within this context, in the past year researchers have developed and published multiple models for COVID-19 prediction using audio [8–13]. Advances in machine learning have demonstrated the potential of automated auscultation of respiratory sounds and brought about new possibilities for fully automated COVID-19 screening [14–21]. For instance, a systematic review by Wynants et al. [22] reported that AUC-ROC (Area Under the Receiver Operating Characteristics Curve) performance of over 75 existing COVID-19 prediction models is in the range of 0.70 and 0.99. Studies in [21, 23] also demonstrated that the motion of the vocal folds during voice production was adversely affected in COVID-19 patients with impairment of respiratory functions, implying that discriminative signatures might be extracted from voice to detect COVID-19.

There is, however, a lack of studies exploring the biases and model evaluation processes that affect (potentially, even positively, but unrealistically) these performance results. Such issues include:

- Potential underlying data biases or study limitations not reported sufficiently, where models were developed and evaluated with limited data which might not be representative of the target population (e.g. 19 subjects in ref. [21], 51 subjects in ref. [24] and 88 subjects in ref. [17]).
- Risk of model overfitting, especially when deploying complex modelling strategies (e.g. a 100% accurate diagnosis of asymptomatic COVID-19 individuals was reported in ref. [16]).
- Methodological flaws (e.g. same users during model development and validation [25]), which would be unrealistic in a practical clinical setting, resulting in an artificial performance boost.
- Lack of systematic comparison with other respiratory diseases like asthma and bronchitis, and only distinguishing COVID-19 from healthy controls [26].

Due to these issues, many researchers raised concerns about the feasibility and effectiveness of such models if deployed in real settings [22, 27, 28].

In this work, we investigate the limits of audio-based COVID-19 testing to create the foundation of realistically applicable audio tools. The aim of this study is two-fold: first, to investigate the performance of an audio-based COVID-19 testing method while addressing the issues noted in the previous studies, by using a large crowd-sourced dataset, to the best of our knowledge, unbiased data, with a methodological design based on realistic assumptions (e.g. independent user split). Second, to explore the impact of biases and design pipeline on the performance.

For this purpose, we first gathered crowd-sourced respiratory sound data from the general population via smartphones. After that, we developed a deep learning model on a portion of the data and then validated its predictive performance on an independent population. In particular, we adhered to the TRIPOD reporting guideline [29], aiming at reporting in a complete, transparent, and usable manner. Our discussion explores the biases and how machine learning model hyperparameters could be tuned, depending on the use of the tool (e.g. on symptomatic or asymptomatic populations) and public health needs.

Our study considerably extends the existing audio-based machine learning research for COVID-19 detection. Our contributions can be summarised as follows:

- We conducted large-scale data collection in real life, covering a wide range of demographics, to study the effectiveness of an audio-based testing tool for COVID-19. Among these gathered data, we carefully selected eligible data to construct the participant sets for model development and evaluation, and responsibly investigated the practical applicability of audio-based COVID-19 detection.
- We explored the realistic performance of audio-based digital testing for COVID-19. Performance of an audio-based model was evaluated systematically and rigorously: we reported the results on various population subgroups divided based on gender and age, presented consistent performance on various COVID-19 prevalence levels, and demonstrated the robustness of the model with respect to confounding factors caused by pathological changes from conditions such as asthma and smoking.
- We explicitly studied the impact of biases and an unrealistic design pipeline. We artificially regrouped the data adding various biases or purposefully including the data from the same participant in both training and testing sets, and showed how the performance in these cases was positively, but unrealistically, affected.

## Results

### Dataset

**Fig. 1:**
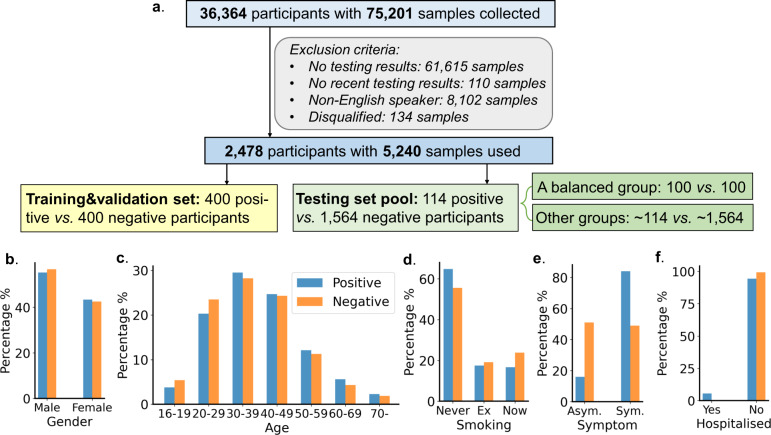
Data flow diagram and demographic statistics. **a** Data cleaning and selecting with 514 COVID-19 positive and 1,964 COVID-19 negative participants eventually included for experiments. **b**–**e** Demographic statistics for the included 2478 participants with blue representing positive class and orange -- negative class:﻿ **b** Gender distribution with about 56% male, 43% female and 1% preferring not to say both two classes. **c** Age distribution with more than 87% positive and negative participants aged 20-59. **d** Smoking history with more than half participants never smoking and the remaining participants having smoked before or smoking currently. **e** symptom distribution: 16% of the positive group showed no symptoms, while 49% of the negative group reported at least one of the symptoms. **f** Hospitalisation: among all the tested participants, only 29 positive and 10 negative participants reported to be in a hospital.

For data gathering purposes, an app (https://www.covid-19-sounds.org) was developed to crowd-source participants’ demographics, medical history, symptoms, COVID-19 test results and audio recordings: three voluntary cough sounds, three to five breathing sounds, and three speech recordings where the user was asked to read a specific sentence. The user had an option to either input that they had done a COVID-19 test and received a positive/negative result, or that they had not been tested at the time of submitting the audio sample. The app is a multi-language tool, but in this study we focus only on audio samples from English-speaking participants (77.7% of the overall number of participants) to avoid language-related bias. Audio quality checks were conducted to filter out incomplete or noisy samples. Finally, 2478 participants (514 positive and 1964 negative) with 5240 samples were included for experiments, as shown in Fig. 1a (more detailed data selecting criteria can be found in Methods section.

Demographic statistics for the experimental data are presented in Fig. 1b–f: 56% of participants in the selected data were male, the majority aged 20–49, half never smoked. In addition, as shown in Fig. 1e, 84% of the participants who tested positive reported symptoms like fever or cough, while others did not report any symptoms at the recording time. 51% of the negative participants reported no symptoms, while 49% had symptoms such as dry/wet cough, fever, dizziness, etc. We also checked the hospitalisation status of the selected participants in Fig. 1f: only 2.6% of the positive and 0.5% of the negative participants were hospitalised at the time of submitting the audio sample. Since over 99% of the samples used in this work came from non-hospitalised participants, the data is adequate to evaluate the developed model in a non-inpatient setting.

### Study design

For machine learning, bias in data will be passed to the mode, leading to wrong predictions, which is especially dangerous in healthcare applications. To explore the realistic performance of audio-based digital testing of COVID-19, in this study we trained deep learning models with audio data collected in the wild. Specifically, we aim to answer the following two research questions:

- RQ1: what is the realistic performance that an audio-based COVID-19 prediction model can achieve?
- RQ2: what is the potential effect of bias that might be introduced in the audio-based COVID-19 detection model?

To this end, we evaluated different models, obtained on either (1) unbiased data or (2) data with purposefully added age or gender bias. We provided a comprehensive analysis and presented our efforts in eliminating potential biases and developing trustworthy COVID-19 testing via sound.

### COVID-19 detection performance

**Fig. 2:**
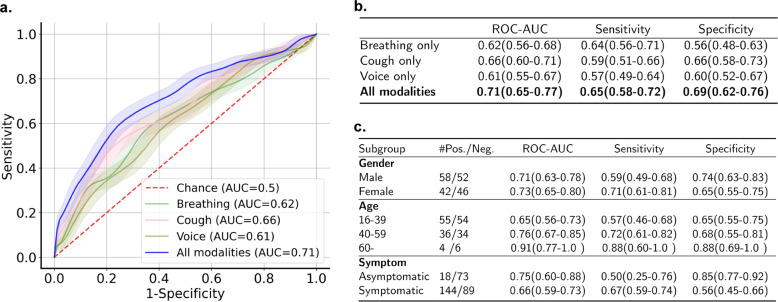
Model performance. **a** Receiver-operating characteristic curve for the binary classification task of diagnosing COVID-19. **b** ROC-AUC, sensitivity and specificity with 95% confidence intervals in brackets for the combination of all modalities or each single modality separately. **c** Subgroup performance comparison under three modalities. For gender and age group, *#* denotes the number of unique positive/negative participants. Note that some participants provided multiple samples, which could be either asymptomatic or symptomatic.

On the demographic-representative testing set with 200 participants (see the age and gender distribution in Supplementary Table 1), our deep learning model with three sound types yielded a ROC-AUC of 0.71 (95% confidence interval (CI) 0.65–0.77) (Fig. 2a), with sensitivity 0.65 (0.58–0.72) and specificity 0.69 (0.62–0.76) (Fig. 2b). The combination of three sound types outperformed any single modality: a ROC-AUC of 0.66 (0.60–0.71) on cough, 0.62 (0.56–0.68) on breathing, and 0.61 (0.55–0.67) on voice was achieved. Moreover, breathing yielded the highest sensitivity of 0.64 (0.56–0.71), but cough showed the highest specificity of 0.66 (0.58–0.73). This indicates that all modalities are informative, and their combination leads to the optimal performance. We further tested the performance of the model on different demographic subgroups under this testing set (Fig. 2c), which shows similar results across different age and gender distributions: ROC-AUCs were all above 0.65, and sensitivity and specificity were similar for each group. Accuracy on the over-60 subgroup is slightly higher, but we suspect that the increased performance might be a result of the limited number of participants in this group. We also inspected how symptoms impact the model performance by dividing the 200 participants in the testing set into asymptomatic and symptomatic subgroups. As presented in Fig. 2c, for both subgroups our model yielded ROC-AUCs above 0.66. Yet, from the comparison we can also observe that our model performs better on distinguishing asymptomatic negative participants (specificity=0.85 (0.77–0.92)) and symptomatic positive participants (sensitivity=0.67 (0.59–0.74)). While for more challenging cases, i.e. predicting symptomatic negative and asymptomatic positive cases, we achieved a lower accuracy: Sensitivity of 0.50 (0.25–0.76) for asymptomatic and specificity of 0.56 (0.45–0.66) for symptomatic participants. A potential explanation could be that asymptomatic positive participants might not manifest changes in audio characteristics, and thus are intractable for detection. Further discussion about the real implications and applications can be found in Discussion section.

### Model performance on the varied prevalence rate

**Fig. 3:**
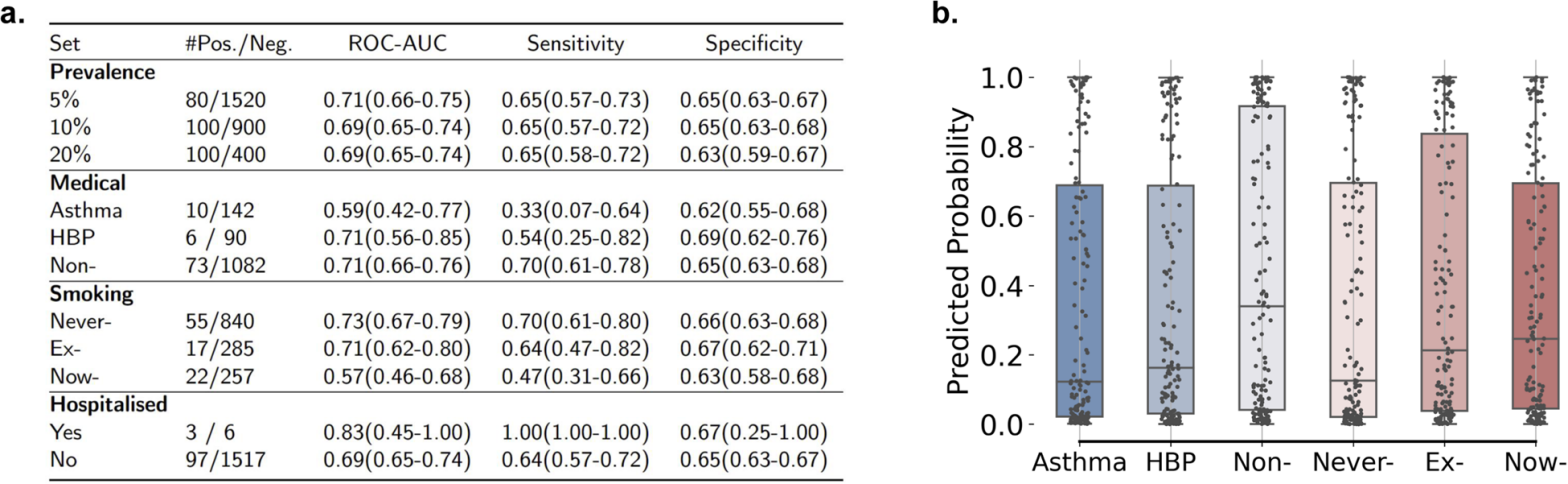
Performance comparison under different conditions. **a** Performance for different prevalence levels, for participants with asthma/HBP or without any medical history, for never-, ex- and now-smoking participants, and for hospitalised and non-hospitalised participants. *#* denotes the number of positive/negative participants. **b** Predicted probabilities of infecting COVID-19 on those negative participants, with values above 0.5 indicating a false positive. No significant difference was observed across subgroups.

According to a recent statistical study, the prevalence rate of COVID-19 ranges from 0.12% to 33% worldwide [30]. Therefore, in addition to testing our model in a balanced setting (50% prevalence level, Fig. 2), we evaluated its performance in various prevalence scenarios. To simulate this, we re-sampled participants from the testing pool (Fig. 1a) and lowered the proportion of COVID-19 positives to 5%, 10% and 20% (Fig. 3a). The performance does not degrade compared to that of 50% prevalence (Fig. 2b): ROC-AUC of 0.71 (95% CI 0.66–0.75), 0.69 (0.65–0.74) and 0.69 (0.65–0.74) can be achieved on 5%, 10%, and 20% prevalence levels, respectively. This is a promising result, suggesting the potential of AI-enabled COVID-19 screening in the real world.

### Model performance on various health and smoking status

One of the most important concerns to address is whether these audio models for COVID-19 testing might be confusing COVID-19 with other illnesses or respiratory pathologies. To investigate this topic further, we split the participants of the testing pool into several non-overlapping groups. Results are presented in Fig. 3.

The first controlled criterion is medical history. We selected participants who reported that they have Asthma, HBP (High Blood Pressure), and those who claimed to have no medical history. We compared the model performance and found that all metrics reach comparable level of accuracy on average: the specificity on Asthma group was 0.62 (0.55–0.68), on HBP group was 0.69 (0.62–0.76), on no medical history group was 0.65 (0.62–0.76) (Fig. 3a) and on a mix of participants was 0.69 (0.62–0.76) (Fig. 2b). Predicted COVID-19 probabilities of the negative participants based on our model are compared in Fig. 3b. Kruskal–Wallis H Test [31] on the three negative groups’ probabilities yielded a *p*-value of 0.62 (>0.05), showing that the predictions are from the same distribution. This validated the assumption that the medical history cannot confuse our model. Worth noting, that the declined sensitivity for Asthma and HBP groups might be caused by the very limited number of testing samples, leading to relatively large performance fluctuations.

The second controlled criterion is the reported smoking status. The variance of the performance across groups was marginal (Fig. 3a): Specificity for those never smoking was 0.66 (0.63–0.68), for those having quit smoking was 0.67 (0.62–0.71), and for those smoking currently was 0.63 (0.58–0.68). Similar to medical history, predicted probabilities for these three are presented in Fig. 3b, with a *p*-value of 0.51 (>0.05) from the Kruskal–Wallis H Test. Sensitivity for smokers was slightly lower: 0.47 (0.31–0.66), which might be explained by the fact that five of the 22 COVID-19-positive smokers were asymptomatic (23% in this group against 16% in Fig. 1e). As our model is better in predicting symptomatic COVID-19 correctly, this explains the slight drop in the overall sensitivity for this group.

Finally, we assessed the performance of hospitalised and non-hospitalised subgroups, separately. From Fig. 3a, our model correctly predicted all positive hospitalised cases (Sensitivity of 1.00) and detected four of the six negative hospitalised cases as COVID-19 negative (Specificity of 0.67). For the majority non-hospitalised group, our model yielded a ROC-AUC of 0.69 (0.65–0.74). This validates the capability of applying our model for pre-screening: distinguishing non-severe COVID-19 infections from the population.

### Model performance with unrealistic evaluation and biases

To show how the bias and unrealistic experiment design impacts the model performance, we re-selected and purposefully introduced various biases that previous works might have had, to generate another four training and testing sets to attempt to artificially inflate the results. The artificially created biases are as follows: (1) Using sample-level random splits (random-splits for short) instead of participant-independent splits (user-splits for short) for training and testing. (2) Introducing gender bias into the data by selecting 85% of the negative participants as female. (3) Bringing age bias into the negative group. There are two biased groups: selecting all negative participants as those aged over 39 (Group 1) and as those aged under 39 (Group 2). (4) Replacing some English-speaking participants with Italian-speaking participants and making the proportion of Italian-speaking participants relatively higher in the positive group. Details of the data used for comparison can be found in Methods section (Supplementary Figs. 3–5). We trained the model without changing the network structure, and if not specifically mentioned, the results are based on the combination of three sound types (breathing, cough and voice).

**Fig. 4:**
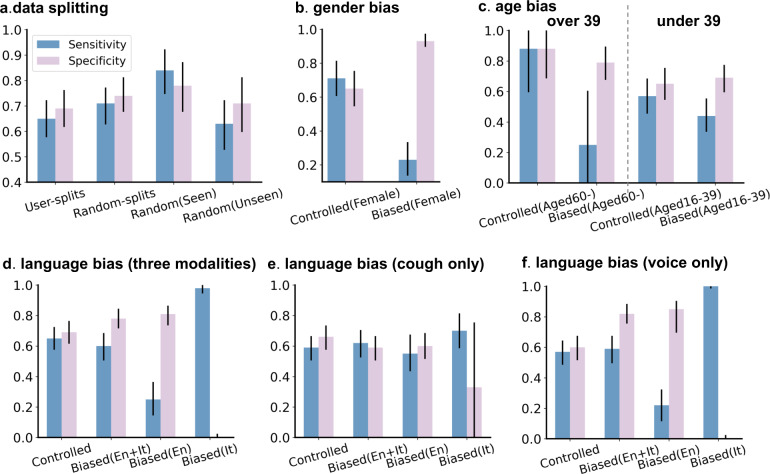
Performance comparison. Sensitivity (blue) and specificity (pink) are presented with sold liner showing the 95% CIs. If not particularly mentioned, the results are based on the combination of three sound types. **a** User-independent splits vs. sample-level random splits: (Seen) denotes the performance on samples whose other samples were used for training, otherwise the performance is notated by (Unseen). **b** Controlled demographics vs gender bias: (Female) denotes the female subgroup. c, Controlled demographics vs two types of gender biases: all negative participants in training set aged over 39 or under 39. (Aged 60-) and (Aged 16–39) denote the elder and the younger subgroup. **d**–**f** Model for English-speakers vs model for biased English- and Italian-speakers: (En) and (It) denote two subgroups from the testing set.

Figure 4 presents the key findings (a detailed comparison can be found in Methods section (Supplementary Tables 2–6). From Fig. 4a, random-splits yielded a higher accuracy than user-splits, with the performance gains coming from the overlapping participants whose data have been seen from training: with the sensitivity of 0.84 (0.75–0.92) and specificity of 0.78 (0.68–0.87), since personal sound traits are easy to memorise for the model. However, this is less realistic, as in real-world scenarios the model should ideally be well adapted to unseen new population. This also may validate our hypothesis that some previous works reported optimistic performance by using this random-split protocol. Demographic bias either in age or gender appears to also lead to biased results. Overall ROC-AUC might be boosted as shown in Supplementary Tables 3 and 4, but a great difference between sensitivity and specificity can be observed in some subgroups. For instance, the sensitivity of 0.23 (0.14–0.33) but specificity of 0.93 (0.90–0.97) were obtained as shown in Fig. 4b on biased (Female) group, because positive females were under-represented in the training set and this model tends to treat female participants as negative. Similar results can be observed in age-biased groups (see Fig. 4c). In the group where negative participants aged-over-39 in training set, the model yielded higher specificity than sensitivity on the aged-over-60 participants in the testing set. On the contrary, the model trained from the data biased to aged-under-39 negative participants yielded higher specificity on the younger group (see Fig. 4c). When it comes to the language bias, i.e., for Italian-speakers, positive participants were over-represented for training, we get the results that sensitivity is as low as 0.25 (0.15–0.36) in English subgroup and specificity is close to 0 in Italian subgroup from Fig. 4d, and this bias particularly impacted voice modality (see Fig. 4f) and slightly influenced cough (see Fig. 4e). Yet, our performance (namely controlled model in Fig. 4) shows consistent sensitivity and specificity across all subgroups, presenting a realistic value for model application.

## Discussion

For digital technologies to penetrate the clinical practice it is pivotal that studies become more explainable and that the models are resilient to the data noise, variability and bias present in real data. Unlike other studies, we highlight the need for more realistic evaluation and report model performance when considering the following factors.

The first is demographic bias. In our study design and data selection, we concerned ourselves with potential confounding factors and tried to rule out selection bias, as these may lead to unrealistically inflated results. Specifically, we split positive and negative samples into three partitions for model training, optimisation and testing, while adjusting the data partitioning and maintaining similar distributions of age and gender across different data splits to control for potential confounding variables (see Supplementary Table 1). This is different from some prior studies in the literature, in which the selection of the data is unclear and lacks a cohort diagram [16, 17]. More importantly, we further performed experimental analysis to explore the effect of demographic bias on the model.

Language bias is another factor that we considered. With the potential of COVID-19 digital testing to be applicable worldwide, it is important to explore the effect of language bias on different audio-based data (such as cough, breathing and voice). To disentangle the possible confounding effect of language, we restricted our analysis to English-speaking data, which gives the most realistic perspective of the capabilities of audio-based diagnostics for COVID-19. In addition, similar to demographic bias, we carried out experiments to test the effect of language bias when the model was trained with unbalanced multi-language data.

Data splitting for experiments also has a notable impact on results. Moreover, in some prior studies, cross-validation was applied for performance validation: this is generally done when the data is scarce and user samples become very important. Data from the same participant might be used for both model training and validation [25, 32]: while this might be considered acceptable in testing theoretical machine learning techniques, if a user appears in both training and testing sets, such models typically do not generalise well, making them poorly-suited for a realistic setting. With the luxury of a large dataset, we could choose to perform user-independent validation, where participants’ data used for model validation are not included and unseen during model training. We are confident that this is a more realistic approach, which could inform future in-the-wild audio-based screening.

Several limitations to our work should be acknowledged. COVID-19 is known to often manifest as respiratory symptoms, which are also common for other relatively widespread diseases, as well as among the smoking population. Therefore, we conducted an in-depth analysis to establish whether our model could be influenced by other respiratory pathology. Specifically, we evaluated the ability of our model to correctly identify a COVID-19 infection in participants who indicated Asthma and High blood pressure in their medical history as well (as these are reasonably large cohorts in our data collection) compared to a cohort who indicated no other medical conditions. We also tested the model on participants with a variety of smoking status reported (e.g. few to many cigarettes per day). However, we note that we have not had the opportunity to test against a wider variety of specific respiratory infections, such as influenza or rhinovirus, since they were not prevalent when our data were collected and are difficult to have a reliable ground truth for. It is also worth noting that some symptoms would be non-respiratory related and may have no effect on the respiratory sounds. However, in the current study, we did not evaluate the performance of our model among different groups of participants with respect to their specific symptoms. The association between symptoms (either respiratory or entirely non-respiratory) and their potential influence on human sounds (either directly or indirectly), and thus the caused effects on audio-based COVID-19 testing, can be investigated in future.

Also, as our models did not fully control for all potential confounding factors such as race and have much less number of elderly participants, future studies should investigate these biases. In addition, though in the present study the language was well controlled (all English), it is yet unclear whether and how different types of accents would affect the model, while we lack such information to study this.

Our data is crowdsourced: we rely on the trustworthiness of the responses from individuals, especially with respect to their COVID-19 testing status. Additionally, some noise inevitably occurs in the collected COVID-19 status labels. The causes of the noise are two-fold: (a) the inaccuracy of a RT-PCR test itself may result in incorrectly reported COVID-19 status; (b) the 14-day time interval in the COVID-19 status may introduce some noise, that is, a person testing negative within the past 14 days may contract COVID-19 later and thus possibly report an improper label. It would be better if the audio data were collected on the same day of the RT-PCR test. The scale of the data helps in amortising the noise generated by the crowdsourcing process while, at the same time, shows robustness of the approach to uncontrolled conditions. Our data, while aims to match the cohort to target population as much as possible, lacks clinical validation. Thus, additional external validation should be performed to assess the generalisation of the prediction model before being applied in clinical practice.

**Fig. 5:**
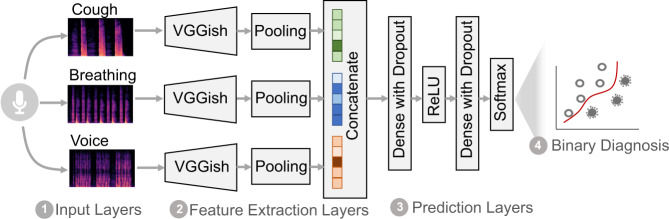
Overview architecture of the deep learning model. A convolutional neural network using cough, breathing, and voice sounds as input, to predict COVID-19 as a binary outcome. *VGGish* is a neural network pre-trained on the Audioset dataset, *Pooling* is an aggregation operator, *Dense* is a fully connected neural network layer, *Dropout* is a randomised operation that reduces overfitting, *ReLU* is a rectified linear unit activation, *Softmax* is the logistic function.

Further, the model’s trainable parameters are optimised based on the probability vector from the final softmax output layer (see Fig. 5): this vector is used to classify COVID-19 positive and negative predictions. While our model could be used on the general population for COVID-19 digital testing, we explore different contexts of applications where the prediction can be adjusted for a more optimal outcome. To achieve this, instead of categorising a participant to the class with the largest probability, we only consider the predicted probability for the positive class and compare it with a threshold. If this value is larger than the given threshold, we will take it as a positive prediction. Hence, we report the ROC curve and sensitivity/specificity under different decision thresholds for asymptomatic and symptomatic groups (participants who did and did not declare symptoms) in Supplementary Figs. 1 and 2, respectively. Specifically, when applying the model with the aim of screening the asymptomatic population for risk of exposure, from Supplementary Fig. 1b), a lower threshold can be used to guarantee a higher Youden Index (defined as Sensitivity + Specificity − 1) and a higher sensitivity compared to the threshold of 0.5, so that potential COVID-19 infections are exhaustively covered, and false positives can be easily filtered by a further clinical testing. Yet, if the targeted group is symptomatic (Supplementary Fig. 2b), to limit the false positives, a higher specificity can be achieved by slightly increasing the threshold to maximise the Youden Index. In this study, as we have limited samples for validation in our dataset, we only demonstrate the performance on the test set data under different threshold settings as a proof-of-concept. For clinical use, a further investigation is required on how to adjust and calibrate this threshold to meet different testing criteria.

As a non-invasive, affordable, and ubiquitous digital screening tool, our model can be applied as an at-home COVID-19 pre-screening tool that is available to the masses to prioritise and allocate the limited clinical resources. In addition to adapting the model to either symptomatic or asymptomatic population, the audio-based predictive models could also be combined with other signatures from other biological signals such as heart rate [33], as well as self-reported symptoms [8] for improved accuracy. Meanwhile, model calibration and uncertainty estimation can be further investigated and incorporated into the automatic diagnosis system, so that the output probability can precisely indicate the confidence of each prediction [34, 35]. Further, when the model is very uncertain towards some inputs, those (a few) samples can be passed to doctors for a clinical test. As such, both testing efficiency and safety can be improved.

In conclusion, we have developed and validated a deep learning method for detecting COVID-19 solely by analysing human sounds via mobile or web applications. In particular, the crowdsourced data have been collected and processed to make the results reliable, by controlling potential confounding factors in COVID-19 positive and negative cases. We analysed the presented model’s predictive performance on detecting COVID-19 infection, which may bring insights into the adoption of digital health technologies in the COVID-19 era. Moreover, we analysed the risks of modelling with various biased data, which led to an overestimated performance. This demonstrated that biased data or modelling should be avoided to rigorously validate the digital testing tool for clinical efficacy.

## Methods

### Data collection

Our data were crowd-sourced via a data gathering framework released in April 2020, in multiple languages and for multiple platforms (a webpage, an Android app, and an iOS app). Collected data consist of participants’ age, gender, medical history, current symptoms11 types of symptoms including headache, fever, dry/wet cough, chills, tightness in your chest, shortness of breath, loss of taste and smell, dizziness, sore throat, runny or blocked nose, muscle aches), and three audio recordings: three voluntary cough sounds, three to five inhalation-exhalation sounds, and the participant reading a standard sentence from the screen three times. Participants were asked whether they had been tested for COVID-19, and an optional geo-location sample was collected. The mobile apps also prompted the participant to input symptoms and sounds every two days. No identifiable information was collected. As of 26th April 2021, a total of 36,364 participants contributed 75,201 samples to our project.

We used samples with self-reported COVID-19 test results for experiments as ground truth. Hence, 61,615 samples without reported test results were excluded. Further 110 samples with COVID-19 testing results declared to be obtained 2 weeks before the recordings were made were also discarded due to the delayed audio recordings with respect to COVID-19 testing. Our data was sourced in multiple languages (English, Italian, Spanish, Portuguese, etc.) and the number of samples in each language varied. To avoid language bias, for the main results of this paper we used English audio samples only, with 8102 non-English samples excluded. Lastly, we manually checked the quality of each recording, deleting in total another 134 samples, that were either incomplete with recordings shorter than 2 s, or samples with silent recordings, or distorted samples with poor audio quality. As a result, 5240 samples from 2478 participants were explored for the majority of the experiments.

### Model architecture

The framework we implemented for COVID-19 classification is a Convolutional Neural Network (CNN) based model, as shown in Fig. 5. The key module, *VGGish*, is a pre-trained CNN, with which we leverage and transfer the knowledge learnt from an external massive general-audio dataset [36].

Specifically, the framework is composed of three parts as below,

(1) Input layers: the network receives one sample with three audio recordings as input, including breathing, cough, and voice from one participant. The audio recording is first chunked into non-overlapping segments of 0.96 seconds. Log-mel spectrogram is computed for each segment with a window size of 25 ms, a window hop of 10 ms, and a periodic Hanning window. 64 Mel bins are adopted for Mel spectrogram covering the frequency range from 125 Hz to 7500 Hz. A small offset is used to convert the mel-spectrogram into log-scale, resulting in a log-mel spectrogram with the size of 64 × 96 per chunk.

(2) Feature extraction layers: the main component of the model is VGGish, a CNN-based network with cascaded convolutional layers, max-pooling and fully connected layers. This network transforms each input spectrogram frame into a 128-dimensional feature vector. Then, an average pooling layer is employed to aggregate all frames within one audio recording into one fixed-length latent feature vector. The size of the CNN kernels and the number of hidden states of fully connected layers are kept consistent with the original work [36].

(3) Prediction layers: the resulting latent feature vectors for three modalities are concatenated, and fed into the binary classifier, which consists of two dense layers (the number of hidden states are 96 and 2, respectively) with non-linear ReLU and Softmax activation functions, respectively. The output of the model is a two-dimensional probability vector: corresponding to the probability of being positive and negative, respectively. If not otherwise specified, we made the categorical prediction as the class with larger probability.

### Data and experiments for evaluation

From the 2478 English-speaking participants, we prepared a training and validation set consisting of 800 participants with balanced COVID-19 status and other demographics to optimise the parameters of our deep learning model, as labelled by the yellow box in Fig. 1a. In the training and validation set, we maintained similar demographic distributions in positive and negative groups (see Supplementary Table 1), aiming to minimise the bias of the crowd-sourced data. The rest of the data were used for evaluation, namely testing set pool (green box in Fig. 1a). To inspect the performance in different realistic deployment scenarios, we first held out a balanced testing set with varied demographics, containing 100 positive and 100 negative participants. Furthermore, we randomly selected positive and negative participants from the testing pool to form new groups with the criteria of various prevalence levels (i.e. the proportion of COVID-19 positive people among the whole population), medical history and smoking status to holistically validate our model.

### Additional data and experiments for bias evaluation

Apart from the controlled training and testing data, to simulate the impact of the unrealistic experiment setting and bias, we also prepared some training and testing sets with improper data splitting and various biases (RQ2). Particularly, different data splits were created to investigate the following four inappropriate scenarios: with different samples from the same participant (may) appearing in both training and testing sets, namely random-split; data split with gender bias; data split with age bias; data split with language. To be more specific, the following strategies were followed to generate the data:

- Individual-dependant Splitting: our balanced training set and testing set contained 1000 participants (800 participants for training&validation, 200 for testing) and 1486 samples (1162 samples for training&validation, and 329 for testing), with 1.5 samples per participant on average. Instead of splitting training and testing by participants, for this comparison group, we randomly shuffled all samples and split them into training and testing according to the original ratio (1162:329).
- Gender bias: to simulate the scenario where COVID-19 positive rate is significantly different in different gender groups, which raises the concern that the model is detecting gender instead of COVID-19, we manually selected 500 positive and 500 negative participants from the total 2,478 participants (blue box in Fig. 1) with gender distribution bias. Specifically, 56% of the positive group are male and the rest 44% are female, while in the negative group, females account for 85% and males for 15%. Age demographics are kept balanced and the total number of participants is unchanged, as shown in Supplementary Fig. 3.
- Age bias: with the same approach, for negative participants, we also purposefully selected (1) those aged over 39, and (2) those aged under 39 to simulate the scenarios when participants were not from the whole population. The revised distribution can be found in Supplementary Fig. 4.
- Language bias: rather than using all English speakers, to investigate the effect of language, we replaced some English-speaking participants with Italian-speaking participants. Specifically, we used more positively-tested Italians than negative. As a result, the positive group mainly consists of Italian speakers, indicating the bias that participants who speak Italian are more likely to be COVID-19 positive. The detailed percentage can be found in Supplementary Fig. 5.

### Model training

After preparing the data splits for model training, we pre-processed the audio data before feeding them into the model. In particular, all the collected audio recordings were resampled to 16 kHz and converted to mono channel. Then, these audio recordings were cropped by removing the silence periods at the beginning and the end of the recording, after which each sample was normalised. Parameters of the deep learning model were updated by iterative gradient back propagation by the binary cross-entropy loss function on the training set. Adam was used as the optimiser [37]. The training batch size was 1. The whole framework was implemented by Python 3.6 and Tensorflow 1.15. Model training was done on an Nvidia Quadro RTX 8000 GPU.

To improve the robustness and generalisation ability of our deep learning model, the following techniques were employed:

- Transfer learning: our collected data is relatively small compared to the number of parameters in the proposed deep neural network. In light of this, we harness transfer learning to improve the representing ability. Specifically, VGGish layers are initialised by a pre-train model, which is designed for audio classification task.
- Differential learning rate: both VGGish and dense layers are jointly updated by using our audio data. However, we used a small learning rate for parameter update of the VGGish part of the network, and increased the learning rate 10 times for the dense layers. Specifically, the learning rate was set as 1e-6 for VGGish and 1e-5 for the final dense layers.
- Avoiding overfitting: we utilised learning rate decay (factor = 0.9) and L2-regularisation (penalty coefficient = 1e-6).
- Two-phase training: to use the data more efficiently, we primarily trained the model via training set and identified the best hyper-parameters based on the averaged sensitivity and specificity of the 15th epoch on validation set, and then we merged the training set to fine tune the model until the training performance kept unchanged.

### Performance analysis

Once a learnt model was obtained (trained and optimised on the training and validation sets), performance was then evaluated on the remaining test set for different demographics, prevalence levels and health conditions. Measures of performance included the ROC-AUC, sensitivity and specificity. For all the metrics, we calculated two-sided 95% CIs, using bootstrap re-sampling with 1000 bootstrap samples and replacement [38].

### Ethics

The study was approved by the ethics committee of the Department of Computer Science at the University of Cambridge, with ID #722. Our app displays a consent screen, where we ask the user’s permission to participate in the study by using the app. Also note that the legal basis for processing any personal data collected for this work is to perform a task in the public interest, namely academic research. More information is available at https://covid-19-sounds.org/en/privacy.html.

### Reporting summary

Further information on research design is available in the Nature Research Reporting Summary linked to this article.

## Supplementary information

Supplementary Information

Reporting Summary

## Data Availability

The data is sensitive, as voice can be deanonymised. Anonymised data will be made available for academic research upon requests directed to the corresponding email. Academic institutions will need to sign a Data Transfer Agreement with the University of Cambridge to obtain the data. We have this agreement in place; please contact covid-19-sounds@cl.cam.ac.uk to obtain it. Once the document is signed, we will provide a download access to where the data is stored.

## Code availability

Python code and parameters used for training of neural networks are available on GitHub at https://github.com/cam-mobsys/covid19-sounds-npjDM for reproducibility purposes.
