# Supplementary material for: Sounds of COVID-19: exploring realistic performance of audio-based digital testing
Source: NPJ Digit Med. 2022 Jan 28;5:16. doi: 10.1038/s41746-021-00553-x (PMC8799654; doi:10.1038/s41746-021-00553-x)
Supplement: Supplementary file 1 — Supplementary Information [file 41746_2021_553_MOESM1_ESM.pdf]

Supplementary Information

Supplementary Table 1. Demographics distribution of the training and balanced testing set.

	Positive			Negative		
	Training	Validation	Testing	Training	Validation	Testing
Male	192(54.9%)	29(58.0%)	58(58.0%))	188(53.7%)	31(62.0%)	52(52.0%)
Female	154(44.0%)	20(40.0%)	42(42.0%)	159(45.4%)	19(38.0%)	46(46.0%)
16-29	92(26.3%)	10(20.0%)	22(22.0%)	73(20.9%)	16(32.0%)	21(21.0%)
30-39	94(26.9%)	16(32.0%)	33(33.0%)	97(27.7%)	13(26.0%)	33(33.0%)
40-49	86(24.6%)	13(26.0%)	23(23.0%)	86(24.6%)	10(20.0%)	24(24.0%)
50-59	39(11.1%)	5(10.0%)	13(13.0%)	50(14.3%)	4(8.0%)	10(10.0%)
60-69	17(4.9%)	1(2.0%)	3(3.0%)	18(5.1%)	4(8.0%)	4(4.0%)
70-	6(1.7%)	2(4.0%)	1(1.0%)	7(2.0%)	1(2.0%)	2(2.0%)

A small proportion (5%) of the participants in each set preferred not to answer about their age or gender.

Supplementary Table 2. Performance of subgroups based on the model trained with Random Splits.

Subgroup	# Pos./Neg.	AUC(95% CI)	Sensitivity(95% CI)	Specificity(95% CI)
**Total**	143(161)/139(158)	0.80(0.75-0.84)	0.71(0.63-0.77)	0.74(0.68-0.81)
**Gender**				
Male	76(85)/77(85)	0.81(0.74-0.87)	0.72(0.62-0.81)	0.75(0.66-0.84)
Female	65(74)/62(73)	0.79(0.71-0.86)	0.70(0.61-0.81)	0.73(0.62-0.83)
**Age**				
16-39	69(79)/59(63)	0.82(0.75-0.89)	0.73(0.63-0.83)	0.76(0.65-0.87)
40-59	59(65)/57(70)	0.76(0.68-0.84)	0.66(0.54-0.77)	0.70(0.59-0.81)
60-	10(12)/15(16)	0.86(0.71-0.98)	0.83(0.58-1.0)	0.88(0.69-1.0)
**Overlap**				
Seen	50(62)/59(76)	0.90(0.85-0.95)	0.84(0.75-0.92)	0.78(0.68-0.87)
Unseen	93(99)/80(82)	0.70(0.62-0.77)	0.63(0.53-0.72)	0.71(0.60-0.81)

# denotes the number of unique positive/negative participants and in brackets are the number of samples for corresponding groups (some participants provided multiple samples). The same for Supplementary Tables 3-6.

a.

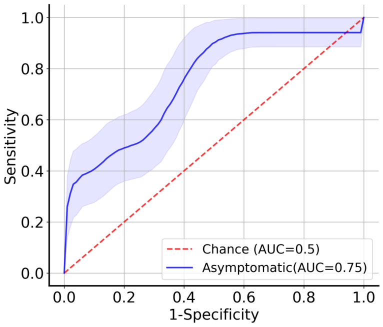

b.

Threshold	Sensitivity	Specificity	Youden-index
0.10	0.89(0.71-1.00)	0.56(0.46-0.67)	0.45(0.24-0.63)
0.15	0.78(0.57-0.95)	0.60(0.49-0.71)	0.38(0.15-0.60)
0.20	0.72(0.00-0.93)	0.62(0.51-0.72)	0.34(0.09-0.58)
0.25	0.67(0.42-0.90)	0.63(0.52-0.74)	0.30(0.04-0.56)
0.35	0.50(0.25-0.76)	0.74(0.63-0.84)	0.24(0.04-0.52)
0.40	0.50(0.25-0.76)	0.79(0.70-0.88)	0.29(0.02-0.57)
0.45	0.50(0.25-0.76)	0.81(0.72-0.89)	0.31(0.03-0.58)
0.50	0.50(0.25-0.76)	0.85(0.77-0.92)	0.35(0.07-0.63)
0.55	0.44(0.19-0.69)	0.85(0.77-0.92)	0.29(0.03-0.56)
0.60	0.44(0.19-0.69)	0.86(0.78-0.94)	0.31(0.05-0.57)
0.65	0.44(0.19-0.69)	0.88(0.80-0.95)	0.32(0.06-0.58)
0.70	0.44(0.19-0.69)	0.88(0.80-0.95)	0.32(0.06-0.58)
0.75	0.39(0.14-0.64)	0.89(0.81-0.96)	0.28(0.03-0.53)
0.80	0.39(0.14-0.64)	0.89(0.81-0.96)	0.28(0.03-0.53)

Supplementary Figure 1. Model performance for asymptomatic screening. a, Receiver-operating characteristic curve for the binary classification task of diagnosing COVID-19. **b,** Sensitivity and specificity with 95% CIs under different thresholds. The default value is 0.50.

a.

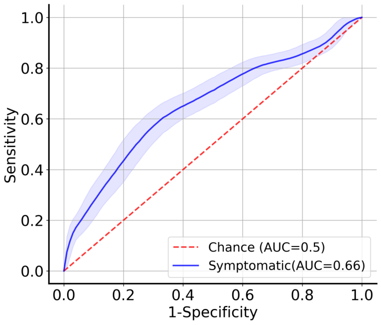

b.

Threshold	Sensitivity	Specificity	Youden-index
0.10	0.83(0.78-0.89)	0.28(0.19-0.38)	0.11(0.01-0.22)
0.15	0.81(0.74-0.87)	0.37(0.27-0.48)	0.18(0.06-0.30)
0.20	0.79(0.73-0.86)	0.40(0.30-0.51)	0.20(0.08-0.32)
0.25	0.76(0.69-0.82)	0.43(0.33-0.53)	0.18(0.06-0.30)
0.30	0.74(0.67-0.81)	0.44(0.33-0.55)	0.18(0.06-0.31)
0.35	0.73(0.65-0.79)	0.49(0.39-0.60)	0.22(0.10-0.35)
0.40	0.71(0.63-0.78)	0.49(0.39-0.60)	0.20(0.08-0.33)
0.45	0.69(0.61-0.77)	0.53(0.42-0.63)	0.22(0.09-0.35)
0.50	0.67(0.59-0.74)	0.56(0.45-0.66)	0.23(0.10-0.36)
0.55	0.65(0.56-0.72)	0.61(0.50-0.70)	0.25(0.12-0.37)
0.60	0.62(0.54-0.70)	0.66(0.56-0.76)	0.29(0.16-0.40)
0.65	0.60(0.52-0.68)	0.67(0.57-0.77)	0.27(0.14-0.39)
0.70	0.58(0.50-0.65)	0.70(0.60-0.79)	0.27(0.15-0.39)
0.75	0.54(0.46-0.62)	0.72(0.62-0.81)	0.26(0.13-0.38)
0.80	0.53(0.45-0.61)	0.73(0.63-0.82)	0.27(0.14-0.38)

Supplementary Figure 2. Model performance for symptomatic diagnosis. a, Receiver-operating characteristic curve for the binary classification task of diagnosing COVID-19. **b,** Sensitivity and specificity with 95% CIs under different thresholds. The default value is 0.50.

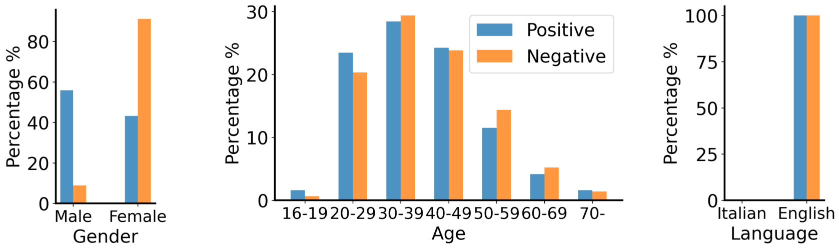

Supplementary Figure 3. Overall Demographic distribution for gender bias evaluation.

Supplementary Table 3. Performance of subgroups based on the model trained with Gender Bias.

Subgroup	# Pos./Neg.	AUC(95% CI)	Sensitivity(95% CI)	Specificity(95% CI)
**Total**	100(162)/100(187)	0.75(0.70-0.80)	0.46(0.38-0.54)	0.90(0.86-0.95)
**Gender**				
Male	58(85)/9(18)	0.62(0.49-0.74)	0.66(0.56-0.76)	0.61(0.36-0.83)
Female	42(77)/91(169)	0.66(0.58-0.74)	0.23(0.14-0.33)	0.93(0.90-0.97)
**Age**				
16-39	55(81)/53(83)	0.69(0.61-0.76)	0.41(0.30-0.52)	0.84(0.76-0.92)
40-59	36(68)/38(86)	0.79(0.71-0.85)	0.56(0.44-0.67)	0.94(0.89-0.99)
60-	4(8)/7(16)	0.96(0.86-1.00)	0.25(0.00-0.62)	1.00(1.00-1.00)

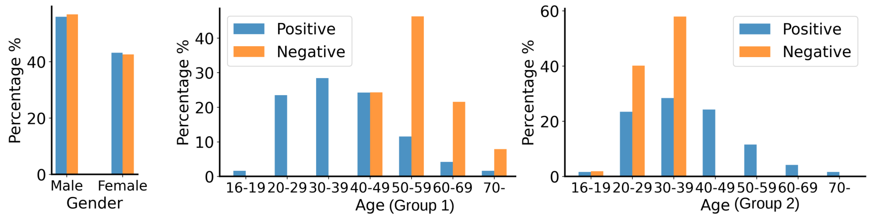

Supplementary Figure 4. Overall Demographic distribution for age bias evaluation.

Supplementary Table 4. Performance of subgroups based on the model trained with Age Bias (Group 1).

Subgroup	# Pos./Neg.	AUC(95% CI)	Sensitivity(95% CI)	Specificity(95% CI)
**Total**	100(162)/100(162)	0.80(0.74-0.84)	0.71(0.63-0.78)	0.73(0.66-0.79)
**Gender**				
Male	58(85)/58(97)	0.81(0.75-0.87)	0.71(0.61-0.80)	0.77(0.68-0.85)
Female	42(77)/42(65)	0.78(0.71-0.85)	0.71(0.61-0.81)	0.66(0.55-0.78)
**Age**				
16-39	55(81)/0(0)	-	0.75(0.65-0.84)	-
40-49	23(41)/25(31)	0.70(0.58-0.81)	0.73(0.59-0.86)	0.61(0.44-0.78)
50-59	13(27)/47(74)	0.74(0.61-0.86)	0.67(0.47-0.84)	0.73(0.63-0.83)
60-	4(8)/28(57)	0.62(0.45-0.79)	0.25(0.00-0.60)	0.79(0.68-0.89)

Supplementary Table 5. Performance of subgroups based on the model trained with Age Bias (Group 2).

Subgroup	# Pos./Neg.	AUC(95% CI)	Sensitivity(95% CI)	Specificity(95% CI)
**Total**	100(162)/100(121)	0.65(0.58-0.71)	0.53(0.45-0.61)	0.69(0.61-0.78)
**Gender**				
Male	58(85)/57(65)	0.64(0.55-0.72)	0.48(0.38-0.59)	0.69(0.58-0.80)
Female	42(77)/43(56)	0.66(0.57-0.75)	0.58(0.48-0.69)	0.70(0.57-0.82)
**Age**				
16-39	55(81)/100(121)	0.60(0.52-0.68)	0.44(0.34-0.55)	0.69(0.60-0.77)
40-	40(76)/0(0)	-	0.63(0.53-0.74)	-

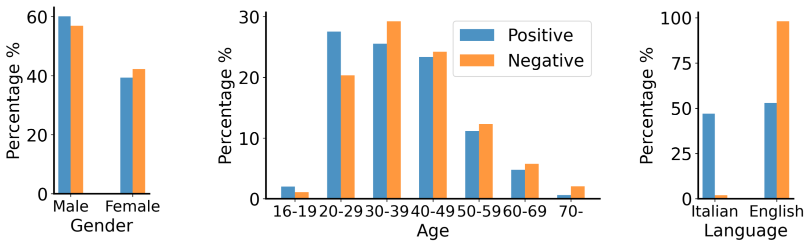

Supplementary Figure 5. Overall Demographic distribution for language bias evaluation.

Supplementary Table 6. Performance of subgroups based on the model trained with Language Bias.

Modality	# Pos./Neg.	AUC(95% CI)	Sensitivity(95% CI)	Specificity(95% CI)
**Three**				
Total	100(139)/100(183)	0.77(0.71-0.82)	0.60(0.51-0.68)	0.78(0.72-0.84)
English	52(73)/98(177)	0.61(0.54-0.69)	0.25(0.15-0.36)	0.81(0.75-0.86)
Italian	48(66)/2(6)	0.42(0.22-0.67)	0.98(0.95-1.0)	0.00(0.00-0.00)
**Breathing**				
Total	100(139)/100(183)	0.55(0.49-0.62)	0.56(0.48-0.65)	0.58(0.51-0.66)
English	52(73)/98(177)	0.53(0.45-0.61)	0.51(0.39-0.63)	0.60(0.53-0.67)
Italian	48(66)/2(6)	0.26(0.06-0.55)	0.62(0.50-0.74)	0.17(0.00-0.57)
**Cough**				
Total	100(139)/100(183)	0.64(0.58-0.7)	0.62(0.53-0.7)	0.59(0.51-0.66)
English	52(73)/98(177)	0.59(0.51-0.68)	0.55(0.44-0.67)	0.60(0.52-0.68)
Italian	48(66)/2(6)	0.57(0.27-0.81)	0.70(0.59-0.81)	0.33(0.00-0.75)
**Voice**				
Total	100(139)/100(183)	0.77(0.71-0.82)	0.59(0.50-0.67)	0.82(0.76-0.88)
English	52(73)/98(177)	0.60(0.52-0.67)	0.22(0.12-0.32)	0.85(0.79-0.90)
Italian	48(66)/2(6)	0.64(0.49-0.77)	1.00(1.00-1.00)	0.00(0.00-0.00)
