# Supplementary material for: Sounds of COVID-19: exploring realistic performance of audio-based digital testing
Source: NPJ Digit Med. 2022 Jan 28;5:16. doi: 10.1038/s41746-021-00553-x (PMC8799654; doi:10.1038/s41746-021-00553-x)
Supplement: Supplementary file 2 — Reporting Summary [file 41746_2021_553_MOESM2_ESM.pdf]

nature portfolio

Corresponding author(s): Tong Xia

Last updated by author(s): Oct 12, 2021

Reporting Summary

Nature Portfolio wishes to improve the reproducibility of the work that we publish. This form provides structure for consistency and transparency in reporting. For further information on Nature Portfolio policies, see our Editorial Policies and the Editorial Policy Checklist.

Statistics

For all statistical analyses, confirm that the following items are present in the figure legend, table legend, main text, or Methods section.

n/a	Confirmed	
☐	☒	The exact sample size (*n*) for each experimental group/condition, given as a discrete number and unit of measurement
☐	☒	A statement on whether measurements were taken from distinct samples or whether the same sample was measured repeatedly
☐	☒	The statistical test(s) used AND whether they are one- or two-sided<br>*Only common tests should be described solely by name; describe more complex techniques in the Methods section.*
☒	☐	A description of all covariates tested
☒	☐	A description of any assumptions or corrections, such as tests of normality and adjustment for multiple comparisons
☐	☒	A full description of the statistical parameters including central tendency (e.g. means) or other basic estimates (e.g. regression coefficient) AND variation (e.g. standard deviation) or associated estimates of uncertainty (e.g. confidence intervals)
☐	☒	For null hypothesis testing, the test statistic (e.g. *F*, *t*, *r*) with confidence intervals, effect sizes, degrees of freedom and *P* value noted<br>*Give P values as exact values whenever suitable.*
☒	☐	For Bayesian analysis, information on the choice of priors and Markov chain Monte Carlo settings
☒	☐	For hierarchical and complex designs, identification of the appropriate level for tests and full reporting of outcomes
☒	☐	Estimates of effect sizes (e.g. Cohen's *d*, Pearson's *r*), indicating how they were calculated

Our web collection on statistics for biologists contains articles on many of the points above.

Software and code

Policy information about availability of computer code

Data collection	Our data were crowdsourced via a data gathering framework released in April 2020, in multiple languages and for multiple platforms (a webpage, an Android app, and an iOS app). Collected data consist of participants' age, gender, medical history, current symptoms, and three audio recordings: three voluntary cough sounds, three to five inhalation-exhalation sounds, and the participant reading a standard sentence from the screen three times. Participants were asked whether they had been tested for COVID-19, and an optional geo-location sample was collected. The mobile apps also prompted the participant to input symptoms and sounds every two days. No identifiable information was collected. As of 26th April 2021, a total of 36,364 participants contributed 75,201 samples to our project.
Data analysis	The whole framework was implemented by Python 3.6 and Tensorflow 1.15.

For manuscripts utilizing custom algorithms or software that are central to the research but not yet described in published literature, software must be made available to editors and reviewers. We strongly encourage code deposition in a community repository (e.g. GitHub). See the Nature Portfolio guidelines for submitting code & software for further information.

Data

Policy information about availability of data

All manuscripts must include a data availability statement. This statement should provide the following information, where applicable:

- Accession codes, unique identifiers, or web links for publicly available datasets
- A description of any restrictions on data availability
- For clinical datasets or third party data, please ensure that the statement adheres to our policy

The data is sensitive as voice sounds can be deanonymised. Anonymised data will be made available for academic research upon requests directed to the

corresponding author. Institutions will need to sign a data transfer agreement with the University of Cambridge to obtain the data. A copy of the data will be transferred to the institution requesting the data. We already have the data transfer agreement in place.

Field-specific reporting

Please select the one below that is the best fit for your research. If you are not sure, read the appropriate sections before making your selection.

☒ Life sciences ☐ Behavioural & social sciences ☐ Ecological, evolutionary & environmental sciences

For a reference copy of the document with all sections, see nature.com/documents/nr-reporting-summary-flat.pdf

Life sciences study design

All studies must disclose on these points even when the disclosure is negative.

Sample size	As our study aim is to develop an audio-based COVID-19 testing machine learning model, we expect as many as data samples that can be used for model learning and evaluating. Yet, audio data collection during pandemic is not easy, and so we continually promote data contribution globally with more than one year. Finally, we use the data collected until we start this work.
Data exclusions	To validate the performance, we exclude samples without COVID-19 testing results, samples without recent testing results, samples from Non-English speakers, and samples that are disqualified.
Replication	Our experiment is carried out by programmers. We keep all the codes to make sure all results are reproducible.
Randomization	This is not relevant, as in our experiment, groups are divided by COVID-19 testing results, which are not random.
Blinding	This is not relevant, as in our experiment, groups are divided by COVID-19 testing results, which are provided.

Reporting for specific materials, systems and methods

We require information from authors about some types of materials, experimental systems and methods used in many studies. Here, indicate whether each material, system or method listed is relevant to your study. If you are not sure if a list item applies to your research, read the appropriate section before selecting a response.

Materials & experimental systems

n/a	Involved in the study	
☒	☐	Antibodies
☒	☐	Eukaryotic cell lines
☒	☐	Palaeontology and archaeology
☒	☐	Animals and other organisms
☐	☒	Human research participants
☒	☐	Clinical data
☒	☐	Dual use research of concern

Methods

n/a	Involved in the study	
☒	☐	ChIP-seq
☒	☐	Flow cytometry
☒	☐	MRI-based neuroimaging

Human research participants

Policy information about studies involving human research participants

Population characteristics	2,478 participants (514 positive and 1,964 negative) with 5,240 samples were included for experiments. 56% participants in the selected data were male, the majority aged 20-49, half never smoked. In addition, 84% of the participants who tested positive reported symptoms like fever or cough, while others did not report any symptoms at the recording time. 51% of the negative participants reported no symptoms, while 49% had symptoms such as dry/wet cough, fever, dizziness, etc.
Recruitment	Participants are volunteered to submitted their data through our developed app. Demographics biases and language imbalance exist in the whole cohort, but we selected balanced subsets for experiments.
Ethics oversight	The study was approved by the ethics committee of the Department of Computer Science at the University of Cambridge, with ID #722. Our app displays a consent screen, where we ask the user's permission to participate in the study by using the app.

Note that full information on the approval of the study protocol must also be provided in the manuscript.
